# Acupuncture for Managing Cancer-Related Fatigue in Breast Cancer Patients: A Systematic Review and Meta-Analysis

**DOI:** 10.3390/cancers14184419

**Published:** 2022-09-11

**Authors:** Tae-Young Choi, Lin Ang, Ji Hee Jun, Terje Alraek, Stephen Birch, Weidong Lu, Myeong Soo Lee

**Affiliations:** 1 KM Science Research Division, Korea Institute of Oriental Medicine, Daejeon 34054, Korea; 2 School of Health Sciences, Kristiania University College, 0107 Oslo, Norway; 3 The National Research Center in Complementary and Alternative Medicine (NAFKAM), Department of Community Medicine, Faculty of Health Science, UiT, The Arctic University of Norway, 9037 Tromsø, Norway; 4 Dana-Farber Cancer Institute, Harvard Medical School USA, Boston, MA 02115, USA

**Keywords:** acupuncture, cancer, breast, fatigue, systematic review, meta-analysis

## Abstract

**Simple Summary:**

Acupuncture (AT) itself is not used to treat cancer. AT is used to help manage various side effects (pain, hot flashes, and fatigue) that occur in cancer treatment. However, the mechanism of action and efficacy of AT are uncertain. The purpose of this systematic review and meta-analysis was to explore the benefits of acupuncture in patients with breast cancer-related fatigue (CRF).

**Abstract:**

Breast cancer (BC) is the most common cancer in women and is a serious threat to women’s health. Cancer-related fatigue (CRF) is a distressing symptom in BC patients during and after chemotherapy or radiation therapy that severely affects quality of life (QoL). AT is widely used for fatigue management. However, the effect of AT on CRF is still uncertain. This study aimed to evaluate the efficacy and safety of AT in the management of CRF in patients with BC. Eleven databases were searched through June 2022. Two researchers independently performed the database search, study selection, data extraction, and risk of bias assessment. Study selection was performed based on predefined Participants, Intervention, Comparators, Outcomes, Study design (PICOS) criteria, and the Preferred Reporting Items for Systematic Reviews and Meta-Analyses (PRISMA) guidelines were followed when reporting the results. A meta-analysis was performed according to the Cochrane systematic review method using RevMan 5.3. A total of 12 studies including a total of 1084 participants were included. The results showed that AT had a beneficial effect compared with sham AT (*n* = 256, SMD = −0.26, 95% CI [−0.51, −0.01], *p* = 0.04, I^2^ = 0%) and a long-term effect on fatigue score (*n* = 209, MD = −0.32, 95% CI [–0.59, −0.04], *p* = 0.02, I^2^ = 0%). Meta-analysis showed that AT had a beneficial effect compared with usual care (UC) on fatigue scores (*n* = 238, SMD = −0.39, 95% CI [−0.66 to −0.12], *p* = 0.005, I^2^ = 0%). Of the 12 articles, 3 articles were judged as having a low risk of bias in all domains and hence were of high quality. No serious adverse effects were identified. AT is an effective and safe treatment for CRF, and AT is more effective than sham AT or UC or wait-list control (WLC). Nevertheless, the methodological quality of most of these studies was low, and the included studies/sample sizes were small, so the ability to derive decisive implications was limited. Further research is needed to confirm these findings.

## 1. Introduction

Breast cancer (BC) is the most common cancer in women and is a serious threat to women’s health [1]. Improved treatment regimens have increased survival rates in recent decades, increasing the number of long-term survivors [2]. Fatigue is a distressing symptom in BC patients during and after chemotherapy or radiation therapy that severely affects QoL [3,4]. At the end of treatment, 40~80% of breast cancer patients report cancer-related fatigue (CRF) [5,6,7]. The exact cause of CRF is unknown. Therefore, how to detect and prevent CRF and effectively treat its side effects remains a challenge.

Unfortunately, accurate and effective pharmacological and nonpharmacologic strategies for managing CRF are lacking. However, exercise, yoga, and meditation seem to be promising treatment options [8]. Currently, BC patients rely on dietary supplements or other alternative therapies without evidence. Acupuncture (AT), one of the common methods of traditional Chinese medicine intervention, has been widely used to treat fatigue.

AT has received increasing attention as an additional strategy for CRF management. The US National Comprehensive Cancer Network (NCCN) guidelines recommend AT for patients with CRF, especially for cancer survivors who have completed anticancer therapy [9,10,11]. Several randomized controlled trials (RCTs) have been conducted to test the efficacy and safety of AT for the treatment of CRF [12]. However, these treatments often require the patient’s subjective cooperation and active participation in activities, the clinical implementation is not strong, and compliance seems to be problematic [13,14].

Clinically, AT is not intended to cure cancer itself but to improve symptoms associated with cancer and cancer treatments [15,16]. The mechanism of action of AT is not clear, but AT has been studied to improve complications such as lymphedema [17,18], hot flashes [19,20], arthralgias (related endocrine therapy) [21,22], and leukopenia (related chemotherapy) [23].

Recently, there has been an increase in systematic reviews (SRs) evaluating the effects of AT on CRF [24,25,26,27]. However, all SRs evaluated the effect of AT not only on fatigue but also on various side effect symptoms. One SR erroneously included a study involving various cancer patients and missed an eligible RCT [24]. Three SRs combined studies regardless of the control intervention of sham acupuncture or usual care (UC); moreover, clinical heterogeneity was present in two SRs [25,26,27], and one SR included various cancers [27]. Additionally, previous studies of the effect of AT on CRF had controversial results. This SR was conducted to identify the effectiveness and safety of AT in managing CRF in breast cancer patients.

## 2. Methods

### 2.1. Study Registration

This protocol has been registered on the Research Registry with the registration number review registry 1254. The Preferred Reporting Items for Systematic Reviews and Meta-Analyses (PRISMA) guidelines were followed when reporting the results [28].

### 2.2. Database and Search Strategy

We searched eleven databases: three international databases (PubMed, EMBASE, and the Cochrane Library), two Chinese databases [CNKI (China National Knowledge Infrastructure) and Wanfang] and six Korean databases [(KoreaMed, OASIS (Oriental Medicine Advanced Search Integrated System), DBpia, KM base (Korean Medical Database), RISS (Research Information Service System) and KISS (Korean Studies Information Services System)] from inception to June 2022. No language limitation was used.

The search terms used were as follows: (“acupuncture” OR “electroacupuncture” OR “electroacupuncture” OR “auricular acupuncture”) AND (“breast cancer”) AND (“cancer related-fatigue” OR “CRF” OR “cancer fatigue”) AND (“randomized clinical trial” OR “RCT”). Additional studies were identified by searching the reference lists of the included studies. The search methods for English, Chinese and Korean databases were similar, and an example of a PubMed search is shown in Table S1.

### 2.3. Inclusion and Exclusion Criteria

#### 2.3.1. Study Design

Only RCTs that used AT to treat CRF were included. Dissertations, theses, guidelines, conference abstracts and narrative reviews were excluded.

#### 2.3.2. Participants

Women with a diagnosis of BC (undergoing chemotherapy/radiotherapy/hormonal therapy) and BC survivors, regardless of cancer stage, who reported fatigue were included.

#### 2.3.3. Interventions

Any type of acupuncture (including electroacupuncture, auricular acupuncture, warm acupuncture, etc.) used as an intervention to treat fatigue in BC (regardless of the number and duration of the treatment) was included. Studies focused on laser acupuncture, acupoint injection, moxibustion or transcutaneous electrical nerve stimulation (TENS) were excluded.

#### 2.3.4. Comparators

We also included trials that compared acupuncture plus conventional treatments (medication, sham acupuncture, UC, or no intervention) versus conventional treatments alone. Studies comparing the efficacy of different acupuncture modalities were excluded. The acceptability of sham acupuncture as a valid control is highly controversial [29,30,31], and we planned to analyze the results using subgroup and sensitivity analyses.

#### 2.3.5. Outcomes

Clinical efficacy (effective rate or cure rate, clinical symptom integral) and fatigue scale scores were used as the primary outcomes. To measure fatigue levels, we considered the following validated tools: the European Cancer Research and Treatment Institution Quality of Life Questionnaire (EORTC QLQ-C30), Piper Fatigue Scale (PFS) and the Multidimensional Fatigue Inventory (MFI).

### 2.4. Study Selection and Data Extraction

All titles and abstracts were evaluated for potentially relevant studies according to the inclusion and exclusion criteria. After screening by titles and abstracts, full-text articles were obtained for further assessment. The data extracted included the author’s first name, publication year, country, sample size, patient age, stage of BC, intervention and control information, outcome measures, the results and adverse events (AEs). The details of intervention were summarized according to Revised STandards for Reporting Interventions in Clinical Trials of Acupuncture (STRICTA) [32]. The selection process and data extraction were independently conducted by two authors (TYC and AL), and any disagreements at each step were resolved through discussion and consultation with a third author (MSL).

### 2.5. Risk of Bias (ROB) Assessment

The Cochrane collaboration ROB assessment tool evaluated seven domains: random sequence generation (selection bias), allocation concealment (selection bias), blinding of participants and personnel (performance bias), blinding of the outcome assessment (detection bias), incomplete outcome data (attrition bias), selective reporting (reporting bias), and other bias. The ROB in each of the above domains was scored as high (−), low (+), or unclear (?). The ROB evaluation was performed independently by two reviewers (TYC and AL). Any disagreements were settled by discussion and consultation with a third author (MSL).

### 2.6. Certainty of Evidence (CoE)

The certainty of evidence (CoE) and the strength of recommendations were assessed using the Grading of Recommendations, Assessment, Development and Evaluation (GRADE) [33]. The level of evidence assessed was classified as high, moderate, low, or very low. The summary of findings table (SOF table) was prepared as a summary of the CoE for each of the major findings obtained through GRADEpro.

### 2.7. Data Analysis

Data analyses were performed using Review Manager version 5.3.5 software (Cochrane Collaboration). The dichotomous data (effective rate) are presented as risk ratios (RRs) with 95% confidence intervals (CIs) and as the mean difference (MD) with 95% CIs for continuous data (fatigue symptom scores). As the variability between the included studies was taken into consideration, the random-effects model was used to pool the data. The chi-square test and Higgins I^2^ test were used to assess heterogeneity. Subgroup analysis was not performed because few studies were included in this review. When missing data were detected, we asked the original study investigators for missing or incomplete information. We assessed the robustness of the results of the meta-analyses by performing sensitivity analyses when appropriate. When possible, publication bias was assessed using a funnel plot.

## 3. Results

### 3.1. Study Identification

**Figure 1:**
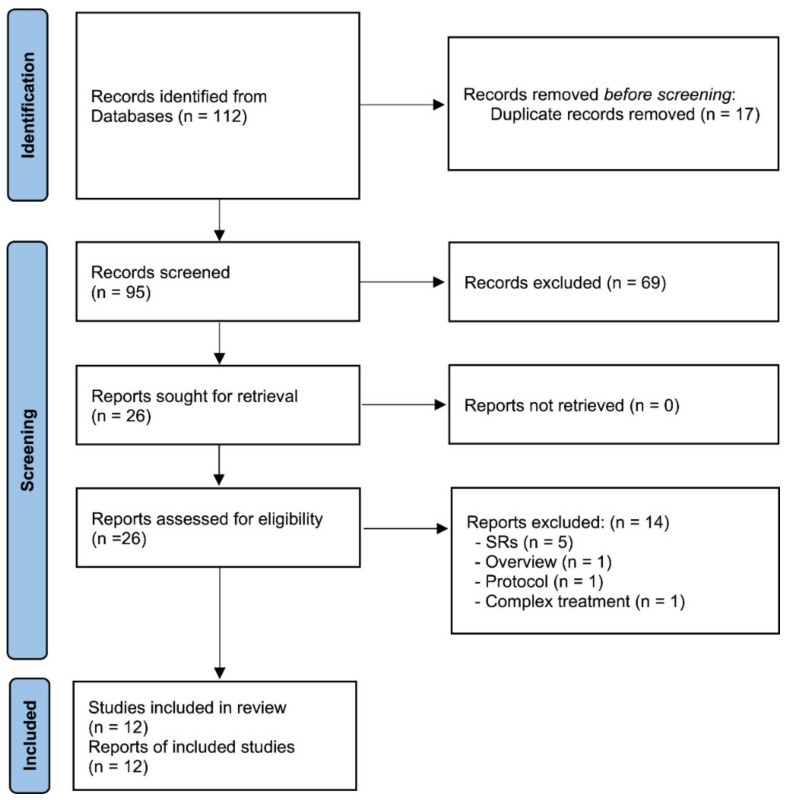
Flow chart of study selection. AT: acupuncture; SRs: systematic reviews.

A total of 112 articles were identified, of which 16 duplicate articles were removed, 84 articles were excluded because they did not meet the inclusion criteria, and 12 studies [34,35,36,37,38,39,40,41,42,43,44,45] were finally selected for analysis, as shown in Figure 1 and Table 1.

### 3.2. Characteristics of the Included Studies

Six RCTs were conducted in China [34,37,38,39,42,45], 2 were conducted in the USA [36,44], 2 were conducted in the UK [40,43], 1 was conducted in Australia [35] and 1 was conducted in Germany [41]; these studies were published from 2011 to 2021, and the sample sizes ranged from 12 to 302 participants (1084 total). Of the 12 RCTs included, 5 were sham controlled, and 7 were open-label trials. A total of 9 RCTs employed a 2-group parallel design, 2 of which compared AT with sham AT control and 7 of which compared AT with UC or WLC. Of those, 3 RCTs employed a 3-group design and compared AT with sham AT, UC or WLC. The study participants were off-treatment in 5 RCTs [35,38,40,43], undergoing chemotherapy in 4 studies [34,39,41,42], undergoing on- and off chemotherapy in 1 RCT [42], undergoing hormonal therapy in 1 RCT [36] and off-surgery in 1 RCT [37]. Most studies used manual acupuncture; only one [36] study used electroacupuncture.

Patients enrolled in the eligible studies received AT once or twice a week for 5 days [45] to 20 weeks [34], and each session lasted anywhere from 20 to 40 min (Table 2). More details are summarized in Table S2 according to STRICTA.

The selection of acupoints was primarily based on the symptoms and syndrome differentiation of traditional Chinese medicine (TCM). After analyzing the points adopted in these tests, we found that ST36 (Zhusanli), SP6 (Sanyinjiao), GV20 (Baihui), CV6 (Qihai) and CV4 (Guanyuan) were the five most commonly used acupoints (Figure S1).

There were eleven studies that used the fatigue score as the effective evaluation standard with continuous data: four studies assessed fatigue using the Brief Fatigue Inventory (BFI) [35,36,39,44], three studies used the MFI [34,40,43], and one study each used the PFS [38], the EORTC QLQ-C30_Fatigue [37], the FACIT-fatigue questionnaire [41] and TCM Symptom Evaluation-fatigue [45].

Three studies [39,42,45] used the fatigue score as the effective evaluation standard with dichotomous data using the effective rate (ER).

### 3.3. ROB Assessment

**Figure 2:**
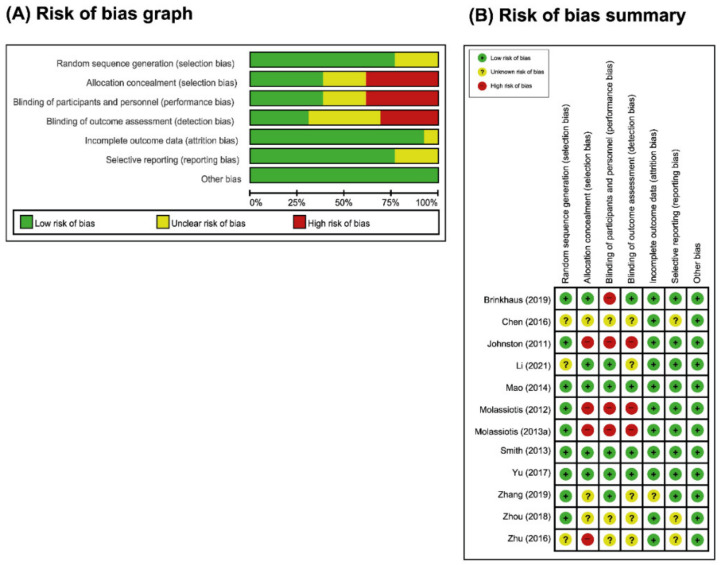
Assessment of risk of bias. (**A**) Risk of bias graph: (**B**) Risk of summary. The present authors’ judgements regarding each item’s risk of bias for all included studies.

For random sequence generation, nine studies [35,36,37,38,40,41,42,43,44] (using a computer-generated random table) were considered to have a low ROB, and three studies [34,39,45] were considered to have an unclear ROB (Figure 2). For allocation concealment, five studies [34,35,36,38,41] were considered to have a low ROB, and seven studies were considered to have an unclear or high ROB. For blinding, five studies [34,35,36,37,38] were assessed as having a low ROB for blinding of participants and personnel and mentioned a sham AT, in which a noninvasive sham needle using the Park device or sham points in a similar setting to the AT groups was used. Except for seven studies included in this review, the nature of the intervention (AT) prevented participants and researchers from being blinded, thus increasing the risk of performance bias. Only four studies [35,36,38,41] were assessed as having a low ROB for blinding of the outcome assessor.

**Figure 3:**
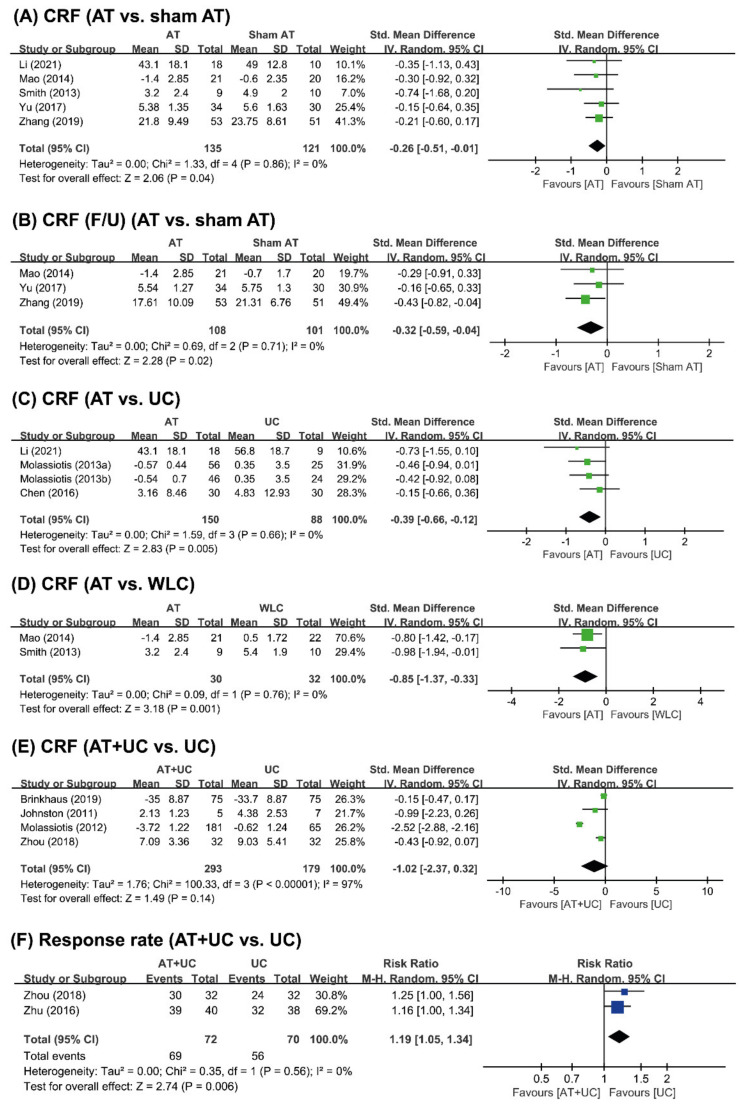
Forest plot of (**A**) AT vs. sham AT for CRF and (**B**) CRF of F/U; (**C**) AT vs. UC vs. UC for CRF; (**D**) AT vs. WLC for CRF; (**E**) AT+UC vs. UC for CRF; and (**F**) effective rate. AT: acupuncture; CRF: cancer-related fatigue; F/U: follow-up; UC: usual care; WLC: wait-list control.

For incomplete outcome data, eleven studies (all but one) reported patient dropouts and had low ROB except one [37]. For selective reporting, nine studies had a low risk of reporting bias [34,35,36,37,38,40,41,43,44], as either the studies were prospectively enrolled or the research protocol was published and the results of the prospectively enrolled were consistent with those published. There was insufficient information to determine this item in three studies [39,42,45], as they lacked a study protocol, and the risk of reporting bias was unclear. For other biases, all RCTs were at low risk of selection bias because the group similarity at baseline was appropriate. Of the 12 studies, 3 studies [35,36,38] were judged to have a low ROB in the major domains (Figure 3A,B).

### 3.4. Outcome Measures

#### 3.4.1. AT vs. Sham AT

Five studies [34,35,36,37,38] compared AT with sham AT. All five studies failed to show the efficacy of AT for CRF. However, meta-analysis showed that AT had a beneficial effect compared with that of sham AT (*n* = 256, SMD = −0.26, 95% CI [−0.51, −0.01], *p* = 0.04, I^2^ = 0%) (Figure 3A). Three studies [37,38] reported the long-term effects of AT on CRF. One study showed favorable effects of AT for CRF compared to sham AT [37], while the other two studies failed to do so [36,38]. The pooled effect showed that AT had a long-term effect on improving CRF (*n* = 209, SMD = −0.32, 95% CI [−0.59, −0.04], *p* = 0.02, I^2^ = 0%) (Figure 3B).

#### 3.4.2. AT vs. UC or WLC

Three studies [34,39,40] compared AT with UC. None of the three studies showed favorable effects of AT for CRF compared with the effects of UC. Meta-analysis showed that AT had a beneficial effect compared with UC on the fatigue score (*n* = 238, SMD = −0.39, 95% CI [−0.66 to −0.12], *p* = 0.005, I^2^ = 0%) (Figure 3C). One study showed beneficial effects on the effective rate [39].

Two [35,36]] compared the effects of AT for CRF with WLC, and both studies reported beneficial effects of AT compared to WLC. Meta-analysis showed that AT was significantly superior to WLC with respect to the fatigue score (*n* = 62, SMD = −2.00, 95% CI [–3.15, −0.86], *p* = 0.0006, I^2^ = 0%) (Figure 3D).

#### 3.4.3. AT+UC vs. UC

Five studies [41,42,43,44,45] compared AT+ UC with UC alone. Two studies showed favorable effects of AT+UC on CRF compared with UC alone [43,44], while the other two studies failed to do so [41,42]. Meta-analysis failed to show superior effects of AT+UC compared with UC alone on CRF (*n* = 442, SMD = −1.02, 95% CI [–2.37,0.32], *p* = 0.14), with high heterogeneity (I^2^ = 97%) (Figure 3E) but positive effects on the effective rate were observed (*n* = 142, RR = 1.19, 95% CI [1.08, 1.34], *p* = 0.0006, I^2^ = 0%) (Figure 3F).

### 3.5. AEs

Three RCTs mentioned AEs of AT in the treatment of CRF. One RCT reported the symptoms of side effects (bruising) [34]. The other two RCTs mentioned side effects, but no AEs were reported [43,44]. Nine RCTs did not report AEs during the study period. There were no AE-related dropouts due to AT in any of the included RCTs.

### 3.6. Certainty of Evidence

According to the GRADE system, the 6 outcomes were ranked as having moderate (*n* = 1), low (*n* = 4) or very low (*n* = 1) quality evidence (Table 3). 

There was unclear or high risk of bias in several studies. Lack of blinding and unclear assignment concealment were identified in several studies. This study was judged to have methodological limitations. Patients, intervention, and comparators provided direct evidence for the clinical question of this review. We judged the evidence to have no serious indirectness. The direction and effect were consistent across studies. We judged the evidence to have no serious inconsistency.

There were significant benefits because the total number of patients enrolled in all studies was small. We determined that the evidence had bounded inaccuracies. The details of the evidence quality assessment are shown in Table 3.

## 4. Discussion

### 4.1. Summary of Main Results

We found that AT has clinically relevant effect sizes in reducing fatigue associated BC when compared to WLC/UC and sham AT. However, the total number of RCTs included and the total sample size were too small, resulting in a limited ability to draw a reliable conclusion about the effectiveness of AT in the management of CRF in BC patients.

Most RCTs were considered to have a moderate or high ROB and consequently limited our ability to elicit clear results on the effectiveness of AT for CRF. The unclear judgments were predominantly in the allocation concealment and blinding of assignments of participants/practitioners/outcomes evaluators because the details were not described. Blinding of participants is important for subjective consequences such as fatigue, but the nature of AT makes it difficult to blind both participants and practitioners. AT includes both the device and the AT process and its techniques, such as needle insertion and manipulation. Achieving proper double blinding is difficult and can cause potential performance bias. The outcome assessor should be blinded to the treatment assignment to reduce detection bias in the study, and the statistician involved in the data analysis is usually blinded to group assignments so that the data can be analyzed and interpreted appropriately without bias.

The safety of AT is still unclear, as only one study [34] reported AEs. However, AT can be considered to some extent a relatively safe treatment if properly performed by a trained acupuncturist. Preliminary findings from one study [46] provide a basis for the idea that AT can be safely used in cancer survivors in an integrated treatment model. A recent study showed that AT is as safe as sham AT and active controls in oncology patients [47].

### 4.2. Overall Completeness and Applicability of Evidence

According to the NCCN guidelines, AT can be used to treat the following cancer-related symptoms: neuropathic pain, arthralgia, myalgia (especially in aromatase inhibitor therapy), nausea, vomiting and fatigue [48]. AT seems to be an effective and safe treatment method for various cancer symptoms and side effects of cancer treatment. A meta-analysis of CRF in several cancer types, including breast cancer, endometrial cancer, gynecologic cancer and other types of malignancy, concluded that AT is effective for CRF management, particularly in breast cancer, which is aligned with our findings [27]. Therefore, AT should be routinely performed in clinical settings to help identify appropriate interventions, treatments, and management strategies to reduce fatigue in cancer patients [49]. Nevertheless, there is a lack of effective therapeutic strategies to treat CRF. AT and related therapies may be considered adjunctive therapy for patients with persistent CRF after primary therapy. Recent research reports that AT may be a useful adjunct for reducing fatigue in cancer palliative care [50,51].

The interpretability of the reported results is also limited due to the large variation in the choice of outcome measures. In some primary studies, fatigue was measured with the BFI, MFI and PFS, but in other studies, the effective rate was reported. In clinical research in China, TCM syndrome evaluation of fatigue and the efficiency rate are commonly used, and fatigue is evaluated by asking patients. Future trials should select the most clinically relevant endpoint as the primary outcome and measure it using validated methods to ensure the utility of future clinical evidence.

Of the 12 RCTs, 11 described the treatment acupoints; manual AT seemed to be more popular than electroacupuncture, and the most commonly used acupoints were ST36 (Zhusanli), SP6 (Sanyinjiao), GV20 (Baihui), CV6 (Qihai), and CV4 (Guanyuan). However, most studies did not investigate the underlying principles of acupoint selection. In this review, the acupoints commonly selected for CRF were the same as those selected in previous reviews [52], which may provide some information for clinical practice. The AT treatment plan, acupoint selection, stimulation methods, AT holding time, and treatment time were different in the included studies, which may affect the results. In addition, the dose of AT is also related to its effectiveness, so other specific factors of AT, such as the course of treatment and frequency of sessions, need to be considered in the future.

Sham AT was adopted as a control in five of the RCTs [34,35,36,37,38]. However, sham AT may not be as suitable as a placebo for assessing the therapeutic effect of real AT [40]. Therefore, identifying appropriate alternative sham control procedures may be a major goal in future AT practice. Several authors have suggested comparative effectiveness studies as a way forward in acupuncture clinical trials [53]. Researchers must ensure rigorous methodologies and adequate reporting to reduce potential bias in studies on the effects of AT on fatigue.

### 4.3. Agreements and Disagreements with Other Reviews

Compared to existing meta-analyses of AT for CRF in BC patients [24,25,26,27], this meta-analysis included five additional newly published RCTs with relatively large sample sizes. We performed a more detailed subgroup analysis according to the clinical heterogeneity determined based on the number of effect sizes included compared to previous researchers who worked on this topic. We also applied stricter/more rigorous inclusion standards to ensure the quality of the source RCTs. Considerable efforts were made to carry out extensive literature searches. Of the 12 studies, 6 were conducted in China, 2 each were conducted in the United States and the United Kingdom, and one each was conducted in Australia and Germany. Therefore, our conclusions are more compelling and can provide a more meaningful reference for use by clinical professionals in developing AT regimens to mitigate CRF in BC patients.

### 4.4. Limitations of the Review

This SR has several limitations. First, we were unable to perform subgroup analysis to identify the magnitude of treatment effects among those CRF patients still undergoing therapy and those who were off-treatment. This subgroup analysis was deemed to be crucial as CRF could occur at any time in the cancer trajectory, and the causes of CRF could be attributed to either the cancer itself or to the cancer therapy. Due to the lack of relevant studies, we could only observe the overall treatment effect of AT for CRF. Second, despite a relatively comprehensive search strategy, incomplete searches of identified studies were inevitable, and we did not include gray literature in this SR. Second, there was an insufficient number of RCTs, and most of the RCTs had relatively small sample sizes. Third, there was significant clinical heterogeneity in the meta-analyses that could not be resolved by subgroup analyses, as such analyses could not be conducted due to the limited number of studies included. Therefore, this study’s results should be interpreted with caution. These limitations may influence the accuracy of the evidence.

## 5. Conclusions

AT is an effective and safe treatment for CRF in patients with BC, and AT is more effective than sham AT, UC or WLC. Nevertheless, the methodological quality of most of these studies was low, and the included studies/sample sizes were small, limiting the ability to draw decisive meaning. Further research is needed to confirm these findings.

## Figures and Tables

**Table 1:** Characteristics of the included RCTs with AT for cancer-related fatigue in breast cancer patients.

Author (Year) [Ref] Country	Sample Size/ Cancer Stage/Current Antitumor Treatment Mean Age (Year)	Intervention (Regimen, Randomized/ Analysed)	Control (Regimen, Randomized/ Analysed)	Fatigue Measurement	Result	AEs/Trial Registration Number
Li (2020) [34] China	40/I to III/ongoing-chemo A: 47.5; B: 42; C: 50.5	(A) AT (1 time weekly for 20 weeks, *n* = 20/18)	(B) Sham AT (noninsertive stimulation at non-AT points, *n* = 10/10) (C) UC (*n* = 10/10)	MFI-20	A vs. B: MD −5.90 [−17.43, 5.63], *p* = 0.32; A vs. C: MD −13.70 [−28.50, 1.10], *p* = 0.07	Bruising ChiCTR-IPR-17013652
Smith (2013) [35] Australia	30/NR/off-treatment A: 55.0; B: 53.0; C: 58.0	(A) AT (2 times weekly for 3 weeks and once weekly for 3 weeks, *n* = 10/9)	(B) Sham AT (noninsertive stimulation at non-AT points, *n* = 10/10) (C) WLC (*n* = 10/10)	BFI	A vs. B: MD −1.70 [−3.70, 0.30], *p* = 0.10; A vs. C: MD −2.20 [−4.16, −0.24], *p* = 0.03	NR/ACTRN12610000720011
Mao (2014) [36] USA	67/I to III/ongoing- HT A: 57.5; B: 60.9; C: 60.6	(A) EA (2 times weekly for 2 weeks and once weekly for 6 weeks, *n* = 22/21)	(B) Sham EA (noninsertive stimulation at non-AT points, *n* = 22/20) (C) WLC (*n* = 23/22)	BFI	A vs. B: MD −0.80 [−2.40, 0.80], *p* = 0.33; F/U: MD −0.70 [−2.13, 0.73], *p* = 0.34 A vs. C: MD −1.90 [−3.32, −0.48], *p* = 0.008; F/U: MD −1.60 [−3.14, −0.06], *p* = 0.04	NR/NCT01013337
Zhang (2019) [37] China	104/I to IV/off-surgery A: 45.1; B: 45.6	(A) AT (2 times weekly for 8 weeks, *n* = 53/53)	(B) Sham AT (noninsertive stimulation at non-AT points, *n* = 51/51)	EORTC QLQ-C30_Fatigue	MD −1.95 [−5.43, 1.53], *p* = 0.27; F/U: MD −3.70 [−6.99, −0.41], *p* = 0.03	NR/NR
Yu (2017) [38] China	72/I to III/off-treatment A: 50.2; B: 51.4	(A) AT (2 times weekly for 4 weeks, *n* = 36/34)	(B) Sham AT (noninsertive stimulation at non-AT points, *n* = 36/30)	PFS	MD −0.22 [−0.96, 0.52], *p* = 0.56; F/U: MD −0.21 [−0.84, 0.42], *p* = 0.51	NR/ISRCTN71727232
Chen (2016) [39] China	60/I to IV/ongoing-chemo A: 50.9; B: 51.2	(A) AT (1 session [1 time daily for 10 days], total 2 session, *n* = 30/30)	(B) UC (*n* = 30/30)	(1) BFI (2) ER	(1) MD −1.67 [−7.20, 3.86], *p* = 0.55 (2) RR 1.21 [1.00, 1.46], *p* = 0.05	NR/NR
Molassiotis (2013) [40] UK	197/I to III/off-treatment NR	(A) AT (1 time weekly for 10 weeks, *n* = 65/56) (B) Self AT (*n* = 67/46)	(C) UC (*n* = 65/49)	MFI	A vs. C: MD −0.92 [−2.30, 0.46], *p* = 0.19; B vs. C: MD −0.89 [−2.30, 0.52], *p* = 0.22	NR/NCT00957112
Brinkhaus (2019) [41] Germany	150/I to III/ongoing-chemo A: 51.4; B: 50.6	(A) AT (NR for 6 months, least 6 sessions), *n* = 75/65) + (B)	(B) UC (*n* = 75/55)	FACIT-fatigue	MD −1.30 [−4.49, 1.89,], *p* = 0.42	NR/NCT01727362
Zhou (2018) [42] China	64/I to IV/mix off and ongoing chemo A: 52; B: 50	(A) AT (3 times weekly for 5 weeks, *n* = 32/32) + (B)	(B) UC (*n* = 32/32)	(1) TCM Symptom Evaluation-fatigue (2) ER	(1) MD −1.94 [−4.15, 0.27], *p* = 0.08 (2) RR 1.25 [1.00, 1.56], *p* = 0.05	NR/NR
Molassiotis (2012) [43] UK	302/NR/off-treatment A: 52; B: 53	(A) AT (1 time weekly for 6 weeks, *n* = 227/181) + (B)	(B) UC (*n* = 75/65)	MFI	MD −3.10 [−3.45, −2.75], *p* < 0.00001	None/NCT00957112
Johnston (2011) [44] USA	13/NR/off-treatment A: 55; B: 53	(A) AT (1 time weekly for 8 weeks, *n* = 6/5) + (B)	(B) UC (*n* = 7/7)	BFI	MD −2.25 [−4.41, −0.09], *p* = 0.04	None/NCT00646633
Zhu (2016) [45] China	78/I to IV/ongoing-chemo A: 47; B: 46	(A) AT (1 time for 5 days, *n* = 40/40) + (B)	(B) UC (*n* = 38/38)	ER (TCM Symptom Evaluation-fatigue)	RR 1.16 [1.00, 1.34], *p* = 0.05	NR/NR

AEs: adverse events; AT: Acupuncture; BFI: Brief Fatigue Inventory; Chemo: Chemotherapy; ER: Effective rate; F/U: Follow-Up; HT: Hormonal therapy; MFI: Multidimensional Fatigue Inventory; NR: Not Reported; PFS: Piper fatigue scale; RR: Risk Ratio; RT: radiotherapy; MD: Mean Difference; UC: Usual Care; WLC: Wait List Control.

**Table 2:** Descriptions of the acupuncture interventions.

First Author (Year) (Ref)	Acupuncture Rationale	Names of Acupoints	Response Sought	Needle Retention Time	Treatment Regime (Total Session)/Follow-Up
Li (2021) [34]	TCM	NR	NR	30 min	Once weekly for 20 weeks (20 sessions)/NR
Smith (2013) [35]	TCM	KI3, KI27, ST36, SP6, CV4, CV6	De qi	20~40 min	Twice weekly for 3 weeks and once weekly for 3 weeks (9 sessions)/NR
Mao (2014) [36]	TCM	SP6, ST36	De qi	30 min	Twice weekly for 2 weeks and once weekly for 6 weeks (10 sessions)/12 weeks
Zhang (2019) [37]	TCM	ST36, SP10, CV17, CV12, GV20 CV7, GV16, BL15, BL45, HT5, KI6	De qi	20 min	Twice weekly for 8 weeks (16 sessions)/16 weeks
Yu (2017) [38]	TCM	GV20, PC6, CV6, ST36, SP6	De qi	NR	Twice weekly for 4 weeks (8 sessions)/8 weeks
Chen (2016) [39]	TCM	GV20, HT7, GV4, GB39, SP6, ST36, SP10	De qi	30 min	Once daily for 10 days, rest 2 days, total 2 courses (20 sessions)/NR
Molassiotis (2013) [40]	TCM	LI4, SP6, ST36	De qi	20 min	Once weekly for 10 weeks (10 sessions)/NR
Brinkhaus (2019) [41]	TCM	PC6, ST36, ST44, CV10, CV12, ST42, LI11, LI10, GV20, CV4, CV6	De qi	NR	NR for 6 months (least 6 sessions)/NR
Zhou (2018) [42]	TCM	Sishen, SP6, ST36, LR3, LR5	De qi	30 min	Third weekly for 5 weeks (15 sessions)/NR
Molassiotis (2012) [43]	TCM	LI4, SP6, ST36	De qi	20 min	Once weekly for 6 weeks (6 session)/NR
Johnston (2011) [44]	TCM/clinical experience	KI 3, LI4, SP6, ST36, SP6, SP4, LU7, KI4, EX-HN3, GV20, HT7, KI4, BL62	De qi	30 min	Once weekly for 8 weeks (8 session)/NR
Zhu (2016) [45]	TCM	ST36, SP10, CV4, SP6, BL23, BL19	De qi	30 min	Once for 5 days (5 session)/NR

NR: not reported; TCM: traditional Chinese medicine theory.

**Table 3:** Summary of findings.

Outcomes	No of Studies (Participants)	Certainty of the Evidence (GRADE)	Relative Effect (95% CI)	Absolute Effects (95% CI)
CRF (AT vs. sham AT)	5 (256)	⨁⨁◯◯ LOW ^a,b^	-	SMD 0.26 lower (0.51 lower to 0.01 lower)
CRF (F/U) (AT vs. sham AT)	3 (209)	⨁⨁◯◯ LOW ^a,b^	-	SMD 0.32 lower (0.59 lower to 0.04 lower)
CRF (AT vs. UC)	4 (238)	⨁⨁◯◯ LOW ^a,b^	-	SMD 0.39 lower (0.66 lower to 0.12 lower
CRF (AT vs. WLC)	2 (62)	⨁⨁◯◯ LOW ^b,c^	-	SMD 0.85 lower (1.37 lower to 0.33 lower)
CRF (AT+UC vs. UC)	4 (472)	⨁⨁⨁◯ MODERATE ^a^	-	SMD 1.02 lower (2.37 lower to 0.32 higher)
Response rate (AT+UC vs. UC)	2 (142)	⨁◯◯◯ VERY LOW ^b,d^	RR 1.19 (1.05 to 1.34)	152 more per 1000 (from 40 more to 272 more)

AT: acupuncture; CRF: cancer-related fatigue; F/U: follow-up; CI: confidence interval; RR: risk ratio; SMD: standard mean difference; UC usual care; WLC: wait-list control. ^a^ Downgraded by one level: unclear or high risk of bias; ^b^ Downgraded by one level: small sample size; ^c^ Downgraded by one level: heterogeneity is high; ^d^ Downgraded by two levels: unclear or high risk of bias. GRADE Working Group grades of evidence: moderate certainty (⨁⨁⨁◯): we are moderately confident in the effect estimate: the true effect is: the true effect is likely to be close to the estimate of the effect, but there is a possibility that it is substantially different; low certainty (⨁⨁◯◯): our confidence in the effect estimate is limited: the true effect may be substantially different from the estimate of the effect; very low certainty (⨁◯◯◯): we have very little confidence in the effect estimate: the true effect is likely to be substantially different from the estimate of effect.

## Supplementary Materials

The following supporting information can be downloaded at: https://www.mdpi.com/article/10.3390/cancers14184419/s1, Table S1. Search strategies; Table S2: Descriptions of the acupuncture interventions according to the revised STRICTA; Figure S1: Most frequently used acupoints.
