# Supplementary material for: Acupuncture for Managing Cancer-Related Fatigue in Breast Cancer Patients: A Systematic Review and Meta-Analysis
Source: Cancers (Basel). 2022 Sep 11;14(18):4419. doi: 10.3390/cancers14184419 (PMC9496910; doi:10.3390/cancers14184419)

Table S1. Search strategy

1. PubMed

#1. (“fatigue”[MeSH Terms]) OR (lassitude[MeSH Terms])

#2. ((((((“fatigue”[Title/Abstract]) OR (lassitude[Title/Abstract])) OR (tired*[Title/Abstract])) OR (“weary”[Title/Abstract])) OR (“weariness”[Title/Abstract])) OR (exhaust*[Title/Abstract])) OR (“lacklustre”[Title/Abstract])

#3. #1 OR #2

#4. breast neoplasms[MeSH Terms]

#5. (((((((“breast neoplasms”[Title/Abstract]) OR (“breast neoplasm”[Title/Abstract])) OR (“breast tumors”[Title/Abstract])) OR (“breast tumor”[Title/Abstract])) OR (“breast cancer”[Title/Abstract])) OR (“breast carcinoma”[Title/Abstract])) OR (“mammary tumor”[Title/Abstract])) OR (“mammary cancers”[Title/Abstract])

#6. #4 OR #5

#7. #3 AND #6

#8. (“acupuncture”[MeSH]) OR (“acupuncture therapy”[MeSH]) OR (“acupuncture points”[MeSH]) OR (electroacupuncture”[MeSH])OR (“acupuncture, ear” [MeSH])

#9. (“acupuncture”[Title/Abstract]) OR (“electroacupuncture”[Title/Abstract]) OR (“electro-acupuncture” [Title/Abstract]) OR (“auricular acupuncture”[Title/Abstract]) OR (“scalp acupuncture”[Title/Abstract]) OR (“dry needling” [Title/Abstract])

#10. #8 OR #9

#11. #7 AND #10

2. CNKI

#1. 乳腺肿瘤 OR 乳腺癌

#2. 疲乏 OR 疲劳 OR 疲倦

#3. #1 AND #2

#4. 针刺 OR 针灸 OR 电针 OR 耳针 OR 头针

#5. #3 AND #4

#6. 随机 OR 对照

#7. #5 AND #6

3. RISS, KISS, OASIS, KTKP

#1. 유방암 AND 피로 AND 침

#2. breast cancer AND fatigue AND acupuncture

#3. #1 OR #2

Table S2. Descriptions of the acupuncture interventions according to the revised STRICTA.

First Author (year) (Ref)	Acupuncture Rationale	Details of needling							Treatment Regime (Total session)/ Follow-up	Other components of treatment	Practitioner background	Control or Comparator intervention
		Number of needle	Names of acupoints	Depth of Insertion	Response sought	Needle stimulation	Needle retention time	Needle type				
Li (2020) [34]	TCM theory	NR	NR	NR	NR	Manual	30 min	0.25 mm × (25 mm/50 mm)	Once weekly for 20 weeks (20 sessions)/ NR	NA	Acupuncturists	Sham AT (non-insertive stimulation at non-AT points)
Smith (2013) [35]	TCM theory	NR	KI3, KI27, ST36, SP6, CV4, CV6	0.5-4.0 cun	De qi	Manual	20 ~ 40 min	(0.22 mm/0.25 mm) × (25 mm/40 mm)	Twice weekly for 3 weeks and once weekly for 3 weeks (9 sessions)/ NR	UC	Acupuncturists	Sham AT (non-insertive stimulation at non-AT points) UC
Mao (2014) [36]	TCM theory	NR	SP6, ST36	NR	De qi	TENS	30 min	0.25 mm × (30 mm/40 mm)	Twice weekly for 2 weeks and once weekly for 6 weeks (10 sessions)/ 12 weekss	UC	Acupuncturists (8, 20 years)	Sham AT (non-insertive stimulation at non-AT points) UC
Zhang (2019) [37]	TCM theory	NR	ST36, SP10, CV17, CV12, GV20 CV7, GV16, BL15, BL45, HT5, KI6	0.5-1.5 cun	De qi	Manual	20 min	0.25 mm × (20 mm/40 mm)	Twice weekly for 8 weeks (16 sessions)/ 16 weeks	NA	NR	Sham AT (non-insertive stimulation at non-AT points)
Yu (2017) [38]	TCM theory	NR	GV20, PC6, CV6, ST36, SP6	NR	De qi	Manual	NR	NR	Twice weekly for 4 weeks (8 sessions)/ 4 weeks	NA	NR	Sham AT (non-insertive stimulation at non-AT points)
Chen (2016) [39]	TCM theory	NR	GV20, HT7, GV4, GB39, SP6, ST36, SP10	NR	De qi	Manual	30 min	NR	Once daily for 10 days, rest 2 days, total 2 courses (20 sessions)/ NR	UC	NR	UC
Molassiotis (2013) [40]	TCM theory	NR	LI4, SP6, ST36	0.5-1.0 cun	De qi	Manual	20 min	36 gauge × (16 to 30 mm)	Once weekly for 10 weeks (10 sessions)/ NR	UC	Acupuncturists	UC
Brinkhaus (2019) [41]	TCM theory	NR	PC6, ST36, ST44, CV10, CV12 ST42, LI11, LI10, GV20, CV4, CV6	NR	De qi	Manual	NR	NR	NR for 6 months (least 6 sessions)/ NR	UC	Acupuncturists (30 years)	UC
Zhou (2018) [42]	TCM theory	NR	Sishen, SP6, ST36, LR3, LR5	0.5-1.0 cun	De qi	Manual	30 min	0.25 mm × 25 mm	Third weekly for 5 weeks (15 sessions)/ NR	NA	NR	UC
Molassiotis (2012) [43]	TCM theory	NR	LI4, SP6, ST36	0.5-1.0 cun	De qi	Manual	20 min	36 gauge × (16 to 30 mm)	Once weekly for 6 weeks (6 session)/ NR	UC	Acupuncturists	UC
Johnston (2011) [44]	TCM theory/clinical experience	NR	KI 3, LI4, SP6, ST36, SP6, SP4, LU7, KI4, EX-HN3, GV20, HT7, KI4, BL62	0.5-1.5 cun	De qi	Manual	30min	(32, 34, 38 gauge) × (0.5 to 1.5cun)	Once weekly for 8 weeks (8 session)/ NR	UC	Acupuncturists (20 years)	Self-care training
Zhu (2016) [45]	TCM theory	NR	ST36, SP10, CV4, SP6, BL23, BL19	NR	De qi	Manual	30 min	NR	Once for 5 days (5 session)/ NR	NR	NR	UC

NR: Not Reported; TCM: Traditional Chinese Medicine; TENS transcutaneous electrical nerve stimulation

Figure S1. Most frequently used acupoints.

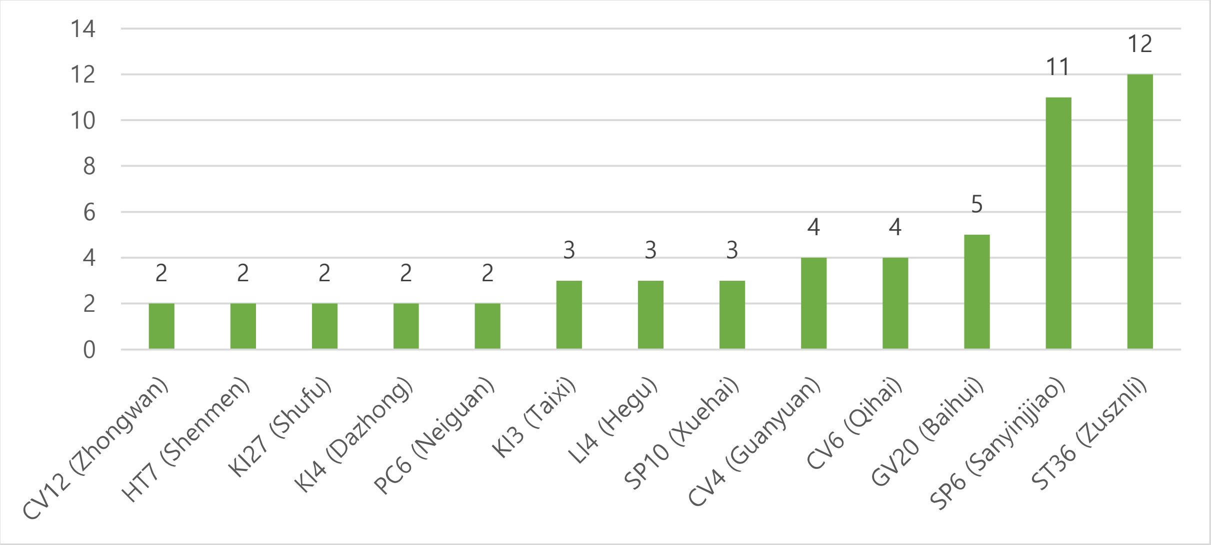

Supplement: Supplementary file 1 [file cancers-14-04419-s001.zip › cancers-1897600-SI.pdf]
